# Bio-Field Array: The Influence of Junction Mediating and Regulatory Protein Expression on Cytoskeletal Filament Behavior During Apoptosis in Triple-Negative Breast Cancer

**DOI:** 10.1177/1178223419830981

**Published:** 2019-02-28

**Authors:** Marcy C Purnell

**Affiliations:** 1 The Loewenberg College of Nursing, University of Memphis, Memphis, TN, USA; 2 Department of Microbiology, Immunology and Biochemistry, College of Medicine, The University of Tennessee Health Science Center, Memphis, TN, USA

**Keywords:** apoptosis, *JMY*, cytoskeletal filaments, autophagy, triple-negative breast cancer

## Abstract

**Introduction:**

The Bio-Field Array (BFA) is a device that generates a dielectrophoretic electromagnetic field (DEP-EMF) when placed in a hypotonic saline solution and a direct current (dc) of ~3 amperes is applied. Human triple-negative breast cancer (MDA-MB-231 cells) is known to have a high percentage of apoptotic (*P53*) deficient cells and some patients can have poor outcomes with current treatments.

**Objectives:**

Previously, we reported a strong upregulation of the apoptotic arm of the unfolded protein response, via reverse transcription–quantitative polymerase chain reaction (RT-qPCR), as well as positive annexin staining in this human breast carcinoma, when grown in media prepared with BFA’s dc DEP-EMF treated saline. Here we will examine and discuss cytoskeletal microtubule changes that were noted in the treated breast carcinoma that are strongly suggestive of apoptosis and the possible correlation of these microtubule changes to the upregulation of Junction Mediating and Regulatory Protein (*JMY*, a *P53/TP53* cofactor) that is known to drive cytoskeleton microfilaments (actin) function.

**Methods:**

In addition to microarray and RT-qPCR analyses, we conducted 7 days of fluorescent microscopic analyses of tubulin assays in these treated versus control MDA-MB-231 cells.

**Results:**

These data suggest 2 possible forms of apoptosis, rounded and irregular, may be occurring and possibly facilitated by the significant upregulation (via microarray and RT-qPCR) of an important but poorly understood Nucleation-Promoting Factor (NPF), *JMY*.

**Conclusions:**

The ability of the BFA dc DEP-EMF to significantly upregulate *JMY* and possibly influence the regulation of unbranched (nucleation-microtubule spikes) and branched forms (autophagy) of actin in the cytoplasmic domains may facilitate a “two coffins” or round and irregular necrosis-like apoptosis for this highly aggressive and often apoptotic-deficient breast cancer cell line.

## Introduction

Triple-negative breast cancers are tumors that lack expression of estrogen receptors (ER), progesterone receptors (PR), and human epidermal growth factor receptor 2 (HER2).^1^ This cancer also represents 15% of all breast cancer cases and occurs more frequently in young Black and Hispanic women than in young women of other racial or ethnic groups.^2,3^ Women with triple-negative breast cancer who do not respond well to endocrine therapy or trastuzumab are noted to have a higher frequency of *P53* gene mutations and often have worse outcomes after chemotherapy than patients with breast cancers of other subtypes.^4,5^

The cytoskeleton of cells is known to perform a multitude of functions that include providing cell shape and mechanical resistance to deformation, stabilization of cell tissues, migration of cells, cell signaling pathway facilitation, uptake of extracellular material through endocytosis, segregation of chromosomes during cell division, and intracellular transport. The 3 major cytoskeletal filaments are microfilaments (actin), intermediate filaments (Ifs), and microtubules and evidence now shows that they all participate in regulating each other to facilitate cytoskeletal function.^6^ Actin is also known to play a role in membrane trafficking while microtubules participate in the control of protrusive and contractile forces.^7^

Junction Mediating and Regulatory Protein (*JMY*) is currently one of the least understood in the Nucleation-Promoting Factors (NPFs) family and is thought to direct transcriptional regulation, cell motility, and trans-Golgi transport in *both* the nucleus and the cytosol.^8^ Scientists have suggested that *JMY*, a *P53/TP53* cofactor, contributes to the assembly of the actin cytoskeleton by initially nucleating new filaments with the unbranched formation of a spire-like (spike) mechanism (independent of Arp2/3) and/or activation of Arp2/3 complex as is seen in branched formations at the autophagosome (autophagy)^9,10^ during apoptosis. Recent studies have shown that microtubules also reorganize during the initiation of apoptosis by forming an apoptotic microtubule network (AMN) that is required to maintain plasma membrane integrity and cell morphology during the process of apoptosis.^11^ The “two coffins” hypothesis also suggests that both AMN and apoptotic cells can display 2 different morphological patterns, round or irregular.^11^ Round-shaped apoptosis is viewed as a physiological and controlled/programmed (transcriptional regulation) type of apoptosis, while irregularly shaped apoptosis can be seen as closer to a necrosis-like death (membrane trafficking).

The Bio-Field Array (BFA) is a device that is currently being researched by a team of nurse scientists along with other scientists and health care professionals.^12–14^ The BFA applies a direct current (dc) driven dielectrophoretic (DEP) electromagnetic field (EMF) to organisms. Here we will analyze how the previously reported significant MDA-MB-231 cell microarray/RT-qPCR (reverse transcription–quantitative polymerase chain reaction) pro-apoptotic gene expression of ChaC glutathione specific gamma-glutamylcyclotransferase1 (*CHAC1*), Tumor Necrosis Factor (*TNFRSF9*), Tumor protein 53-induced nuclear protein 1 (*TP531NP*), Caspase 4 (*CASP4*), along with the pro-survival gene expression of X-box binding protein 1 (*XBP1*) in the MDA-MB-231 cells grown in the BFA-treated media may show a correlation between the noted microtubule assembly (tubulin assay) and possible branched and unbranched actin bundling changes (upregulation of *JMY*), in vitro, in the noted round and possible irregular necrosis-like apoptosis in this highly aggressive human breast carcinoma.^12^

## Materials and Methods

### Cell growth media

Human MDA-MB-231 triple-negative breast carcinoma cells were obtained from the American Type Culture Collection (ATCC). The MDA-MB-231 cells were maintained in high glucose Dulbecco’s Modified Eagles Medium (DMEM, Lonza #12-604Q) containing 10% fetal bovine serum (FBS; Atlanta Biologicals). To prepare dc DEP-EMF treated and control DMEM, 10X DMEM (Sigma cat. #D2429) was diluted 9:1 with a hypotonic saline solution that had been treated for 30 minutes at 2 amperes of dc with the BFA device (time and amperes noted have been determined to be required to obtain sufficient dielectrophoresis and magnetic/electrical behavioral change of ionic concentrations in solution) or with an aliquot of the same solution (control) prior to treatment with the device. The hypotonic saline solution consisted of 3 mM NaCl prepared using laboratory-grade deionized water and molecular biology-grade NaCl (Promega cat. #V4221). Complete treated and control DMEM was supplemented with 0.004 gm/L folic acid (Sigma-Aldrich cat. #F8758-5G), 4000 mg/L glucose (Sigma-Aldrich cat. G7021-100G), 0.584 gm/L glutamine (Sigma-Aldrich cat. #G7513), and 3.7 gm/L sodium bicarbonate (Biowhittaker cat. #15-6131) and filtered through a 0.22-micron pore size bottle top filter (Corning cat. #430624). Fetal bovine serum was then added to 10% final concentration.

For the BFA-treated and control groups, aliquots of 10 000 cells were seeded into three 6-well plates for each of the 2 groups using media purchased from commercial vendors and supplemented with either FBS or horse serum. The next day, the media were replaced with freshly prepared BFA-treated or control media until the cells were removed for tubulin assay, microarray, and RT-qPCR analyses.

### Tubulin staining assay

To quantify and visualize the nucleus and tubulin structure in human breast carcinoma, cells were grown in control or treated media on glass cover slips for the time point analyses of 1, 2, 3, 4, 5, 6, and 7 days. The cells were then fixed and stained at each time point with DAPI to visualize the nuclei and with an anti-tubulin antibody followed by a rhodamine-conjugated secondary antibody. The cells were then examined with fluorescent microscopy.

### Microarray analyses

Replicate 60 mm dishes of MDA-MB-231 (5 plates each for growth in treated and control media) were plated in DMEM-10 and the next day the media were replaced with either treated or control media which was replaced daily with freshly prepared treated or control media for the next 2 days. On day 4 post-plating (day 3 post-treatment), the cells were removed with trypsin, counted, and 3 × 10^6^ cells from each plate were collected by centrifugation and total RNA was isolated using the RNeasy Mini Kit according to the manufacturer’s instructions (Qiagen). RNA concentration was determined and RNA integrity was evaluated using an Agilent 2100 Bioanalyzer (Agilent Technologies) and all RNA integrity number (RIN) values were ⩾10. The RNAs from the 5 biologic replicates from each group were combined and cDNA was generated using Ambion WT amplification kit (ThermoFisher Scientific) according to the manufacturer’s instructions. The samples were subsequently fragmented and labeled using the Affymetrix WT Terminal Labeling kit and then hybridized, together with the probe array controls, onto the Human Genome U133 Plus 2.0 GeneChip Array (Affymetrix). The array was washed and stained using an Affymetrix Fluidics Station 450, scanned on an Affymetrix GCS3000 7G scanner, and the data were normalized by Robust Multichip Averaging (RMA) using the Affymetrix expression console to transform all the arrays to have a common distribution of intensities by removing technical variation from noisy data before analysis. To quantile normalize 2 or more distributions to each other, both treated and control groups were set to the average (arithmetical mean) of both distributions. Therefore, the highest value in all cases becomes the mean of the highest values, the second highest value becomes the mean of the second highest values.

### Quantitative RT-PCR analyses

Junction Mediating and Regulatory protein of *P53/TP53* (*JMY*), *CHAC1, TNFRSF9, TP531NP, CASP4*, and *XBP1* were some of many that showed significant differences in expression between the treated and control MDA-MB-231 groups via microarray analyses and were subsequently validated by RT-qPCR while ribosomal protein S19 (*RIBOPROTS19*) was used for normalization. The primers were designed using the Universal Probe-Library Probe-Finder assay design software (Roche) and sequences are available on request. RNAs from the 5 biological replicates were reverse transcribed individually using the Transcriptor First Strand cDNA Synthesis Kit (Roche) to generate cDNAs according to the manufacturer’s protocol. Ten-fold serial dilutions (10^−1^ – 10^−4^) of each of the cDNAs were then mixed with the appropriate universal library probe (UPL probe, Roche), sense and anti-sense primers, and reaction buffer into 96-well plates. The polymerase was activated by incubation at 95°C for 5 minutes following by a 45-cycle amplification consisting of denaturation at 95°C for 15 seconds, annealing at 60°C for 1 minute and elongation at 72°C for 5 minutes.

## Results

### Tubulin assay

Our previously reported results with the microarray and quantitative RT-qPCR results in the MDA-MB-231 cells suggested that these treated cells showed a strong transcriptional upregulation of the *ATF4-CHOP-CHAC1* apoptotic arm of the unfolded protein response (UPR) and *JMY*, an actin regulator.^12^ As tubulin assembly and actin bundling play critical roles in the execution of apoptosis and to investigate how the mitotic spindle and microtubule formation changes occurred and possibly coincided with these RT-qPCR results, human breast carcinoma cells were grown in control or treated media for 7 days.^15^ The cells were then fixed daily and stained with DAPI to visualize the nuclei and with an anti-tubulin antibody followed by a rhodamine-conjugated secondary antibody. Here we analyzed not only the mitotic spindle formation (mitosis) but the structure and size changes that were noted in the microtubule organization/structure as well as the size of the nucleus and characteristics noted in the nucleus.

**Figure 1.**
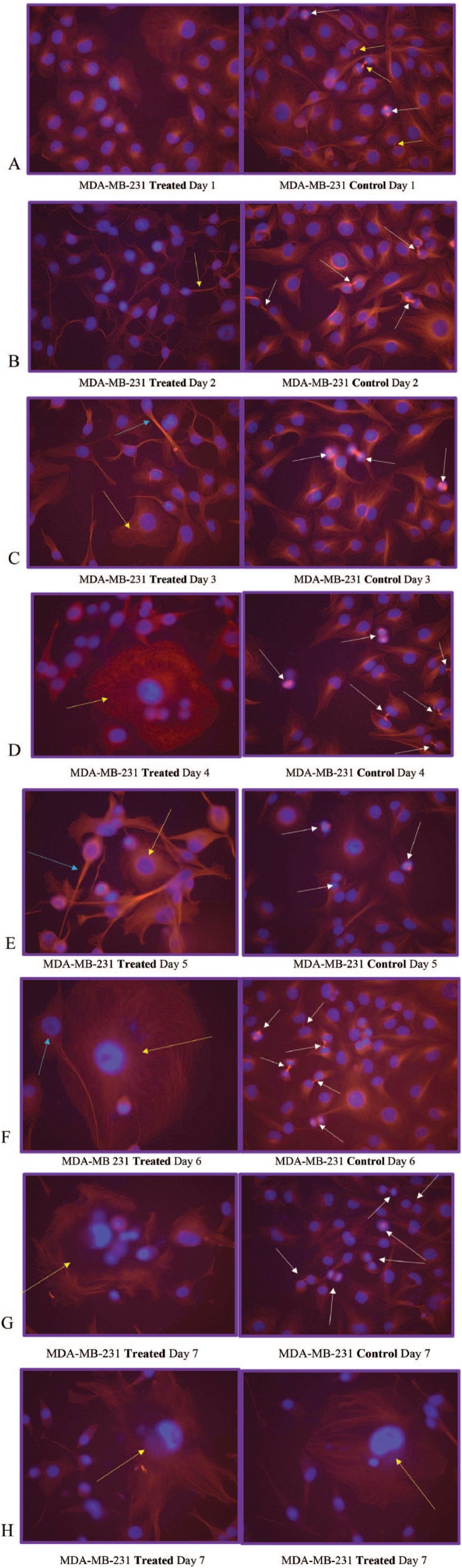
(A) MDA-MB-231 Treated Day 1 and MDA-MB-231 Control Day 1. Note the microtubulin (red) formation structural changes of cell contraction (nucleus is blue) in the treated cells (left) after 1 day of growth in the treated media with no cells seen actively undergoing mitosis. The control cells (right) appear to be actively undergoing mitosis (note the chromosomes in blue lining up at the poles for mitosis [white arrows] and almost complete with 2 daughter cells [yellow arrow]). (B) MDA-MB-231 Treated Day 2 and MDA-MB-231 Control Day 2. The cells in the treated group begin to show initial microtubule spikes (yellow arrow—hallmark of possible apoptosis, caspase driven-cytoskeleton reorganization of tubulin assembly and possible unbranched actin bundling) with no cells noted to be undergoing mitosis. The control cells continue to undergo mitosis (white arrows) with expected tubulin formation. (C) MDA-MB-231 Treated Day 3 and MDA-MB-231 Control Day 3. The cells in the treated group continue to show no mitosis and some cells are noted to microtubule spikes of possible tubulin assembly and possible unbranched actin bundling (light blue arrow) while others appear to have enlarged tubulin structures with possible branched actin bundling (yellow arrow—consistent with possible autophagy) noted in Figure 3. The cells in the control group continue to undergo mitosis (white arrows). (D) MDA-MB-231 Treated Day 4 and MDA-MB-231 Control Day 4. The cells in the treated group continue to not show mitotic activity. The nuclei appear to be blurring/blebbing with cytoskeletal changes (possible round apoptotic microtubule spikes and unbranched actin bundling), enlarged tubulin structures, and nucleus (possible autophagy with possible branched actin bundling—yellow arrow). The control cells continue to actively undergo mitosis (white arrows). (E) MDA-MB-231 Treated Day 5 and MDA-MB-231 Control Day 5. The cells in the treated group continue to not show mitotic activity. The nuclei continue to show blurring/blebbing (undergo possible round apoptosis—light blue arrow) with microtubule spikes along with enlarged tubulin structures and nucleus (possible necrosis-like apoptosis—yellow arrow). The control cells continue to undergo active mitosis (white arrows). (F) MDA-MB 231 Treated Day 6 and MDA-MB-231 Control Day 6. The cells in the treated group continue to not show mitotic activity. The nuclei appear to continue to show blurring/blebbing (rounded apoptosis—light blue arrow) with microtubule spikes and others with considerable tubulin and nuclear enlargement (possible autophagy, nuclear envelope swelling/breakdown—cell death/necrosis-like-apoptosis—yellow arrow). The control cells continue to undergo mitosis (white arrows). (G) MDA-MB-231 Treated Day 7 and MDA-MB-231 Control Day 7. The cells in the treated group continue to not show mitotic activity. The nuclei appear to continue to show blurring/blebbing as well as what appears to be catastrophic swelling/enlargement/breakdown of the microtubulin structure and swelling/breakdown of the nucleus/nuclear membrane (autophagy-necrosis like apoptosis- yellow arrow). (H) MDA-MB-231 Treated Day 7 and MDA-MB-231 Treated Day 7. The cells in the treated groups continue to not show mitotic activity. The nuclei appear to continue to show blurring/blebbing (with round apoptotic microtubule spikes) and enlargement/swelling/breakdown of the nucleus/nuclear membrane (autophagy-necrosis-like apoptosis—yellow arrows).

**Figure 2.**
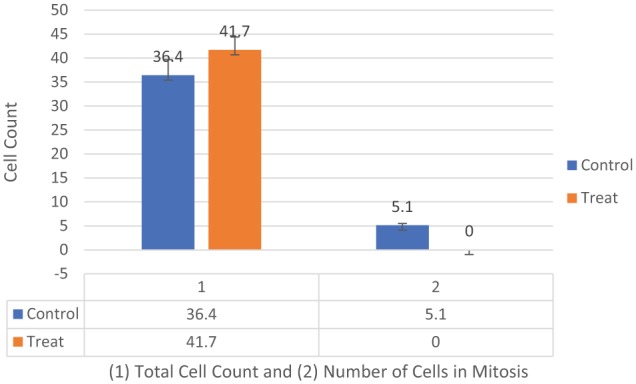
MDA-MB-231 cells tubulin assay total cell count and total number of cells undergoing mitosis in the control versus treated groups (n = 10, 10, 10). Two-way analysis of variance with replication was conducted with significance at (*P* < .05) (Table 1). There was a significant difference between the number of cells undergoing mitosis in the control versus treated groups of MDA-MB-231 cells (*P* = .019545).

**Figure 3.**
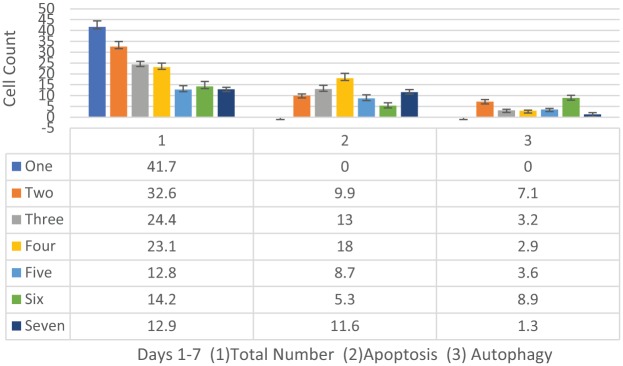
MDA-MB-231 cells tubulin assay (n = 10, 10, 10) total cell counts, number of cells displaying possible apoptosis, and number of cells displaying possible autophagy. The data suggest that there is a decrease in cell count over the 7-day assay, an increase in apoptotic cell numbers from day 2 to 4 that decrease on days 5 and 6 and then increase again on day 7 as the autophagy shows increase on days 2 and 6, with a decrease on day 7.

**Figure 4.**
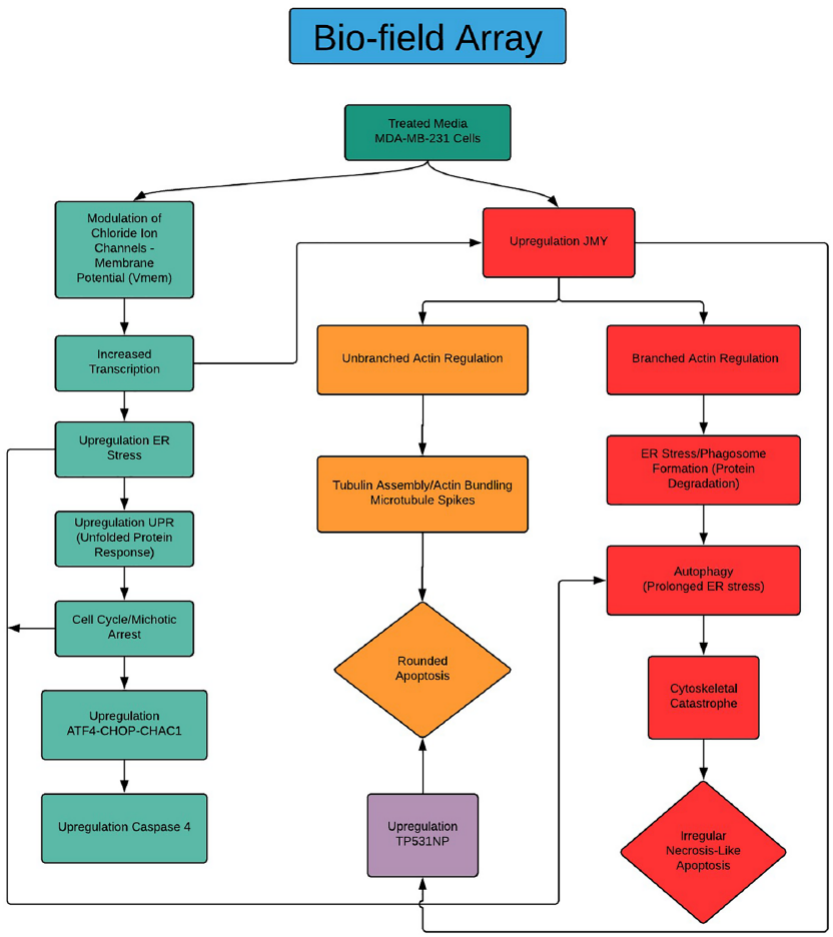
Rounded versus irregular (possible *JMY* cytoplasmic domain influence) apoptosis in MDA-MB-231 cells grown in Bio-Field Array treated growth media. A portion of the treated cell population (non-P53 deficient) may be transitioning into apoptosis from the upregulation of the ER Stress/UPR/TP531NP (rounded/programmed apoptosis with microtubule spikes/unbranched actin bundling) and the *P53*-deficient populations of cells may transition to a more irregular necrosis-like apoptosis due to the prolonged ER stress and the pro-apoptotic survival process (autophagy/phagosome formation) and possibly increased tubulin assembly/*branched* actin bundling that triggers cytoskeletal catastrophe. ER indicates estrogen receptor; *JMY*, Junction Mediating and Regulatory Protein; UPR, unfolded protein response.

**Figure 5.**
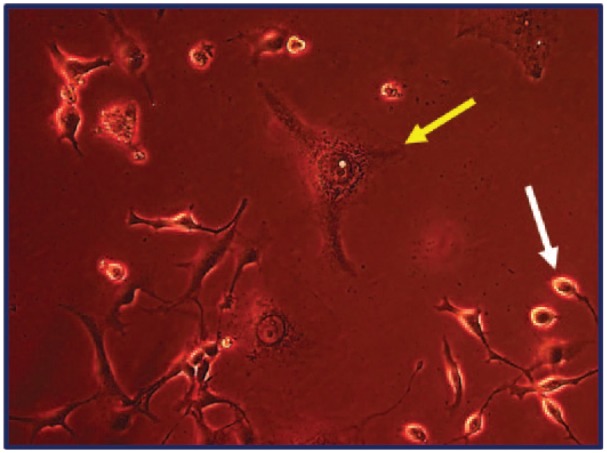
MDA-MB-231 Cells—Initiating morphology consistent with rounded (white arrow) and irregular (autophagy—yellow arrow) apoptosis after 48 hours in treated media.

On day 1 of our analysis, the tubulin formation had some noted structural changes (cell contraction) of early apoptosis in microtubules in the treated cells with no cells seen actively undergoing mitosis while the control cells appear to be actively undergoing mitosis (Figures 1A and 2). On day 2, the cells in the treated group appear to show possible microtubule apoptotic spikes (microtubule reorganization/assembly with possible unbranched actin bundling) with no cells noted to be undergoing mitosis, while the control cells continue to undergo mitosis with expected tubulin formation (Figure 1B). The cells in the treated group on days 3 through 5 continue to show no mitosis and some cells are noted to display possible microtubule apoptotic spikes (rounded apoptosis—transcriptional regulation) (Figures 1, 3, and 4) while others appear to have enlarged and irregular tubulin structures (consistent with possible autophagy-irregular necrosis-like apoptosis; membrane trafficking) (Figures 1C to E, 3, and 5). The cells in control group also continue to undergo active mitosis. On day 6, the cells in the treated group continue to not show mitotic activity. The nuclei appear to continue to show blurring/blebbing with the noted elongation of the tubular reorganization (possible unbranched actin bundling and possible rounded apoptosis) and others showed considerable tubulin and nuclear enlargement in the other possible irregular-necrosis-like cells (possible branched actin bundling and membrane trafficking) (Figures 1 and 3). The control cells continue to undergo active mitosis (Figure 1F). Finally, the cells in the treated group on day 7, continue to not show mitotic activity. The nuclei appear to continue to show blurring/blebbing of possible rounded apoptosis (with microtubule apoptotic spiking) as well as enlarged/swelling/breakdown of the tubulin structure and nucleus (possible irregular necrosis-like apoptosis; membrane trafficking) (Figures 1G, H and 3). The control cells continue to undergo active mitosis (Figure 1G). Finally, there was a significant difference between the treated versus control cells undergoing mitosis when analyzed with a 2-way analysis of variance (ANOVA) with replication analysis (*P* = .019545) (Table 1).

**Table 1.** Two-way ANOVA with replication for total number and mitotic cells in control and treated MDA-MB-231 cells, day 1.

ANOVA						
Source of variation	SS	*df*	MS	F	*P*	F crit
Sample	0.1	1	0.1	0.002209	.96277	4.113165
Columns	13 322.5	1	13 322.5	294.3476	6.55E–19	4.113165
Interaction	270.4	1	270.4	5.974224	.019545	4.113165
Within	1629.4	36	45.26111			
Total	15 222.4	39				

These results would lead us to reject the H_0_ and conclude that there was a significant effect of the BFA dc DEP-EMF on the mitosis of control versus treated MDA-MB-231 cells. Abbreviations: ANOVA, analysis of variance; BFA, Bio-Field Array; DC, direct current; DEP-EMF, dielectrophoretic electromagnetic field.

### Microarray and RT-qPCR

**Figure 6.**
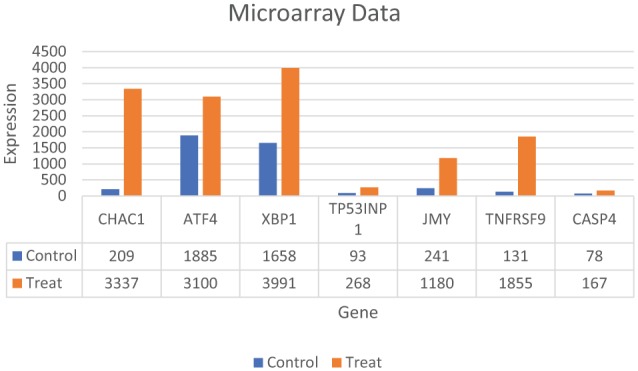
Gene expression profiling of MDA-MB-231 cells grown in treated versus control media. Five replicate plates of the MDA-MB-231 cells were maintained in treated or control media for 3 days with media changes daily. Total RNA was isolated and gene expression profiling was performed using the Affymetrix Human Genome U133 Plus 2.0 Gene Chip. A subset of genes involved in apoptosis and the cell survival (UPR) are shown here.^12^ UPR indicates unfolded protein response.

**Figure 7.**
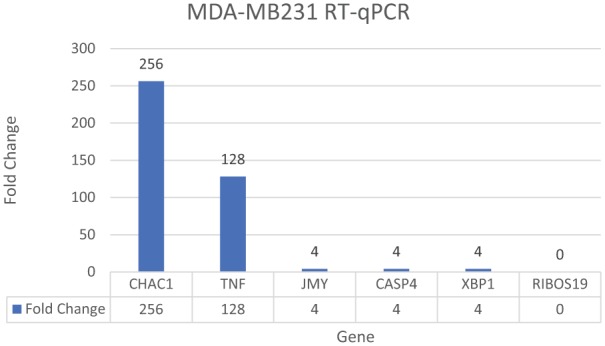
MDA-MB231 cells RT-qPCR at 3 day time point via delta-delta Ct method. *CHAC1* and *TNFRSF9* showed strong upregulation in the treated groups of the MDA-MB-231 cells. *CHAC1* and *TNFRSF9* are pro-apoptotic genes (*ATF4/ATF3/CHOP/CHAC1*) and strongly suggest apoptosis may be occurring in the treated cell population. *JMY* shows upregulation which shows this actin regulator may have increased influence in the actin formation in the treated cell population. *CASP4* is the caspase linked to the UPR and also shows upregulation. *XBP1* shows upregulation and this is linked to the pro-survival arm *IRE1a/JNK/XBP1* of the UPR and shows some of the treated cell population has pro-survival (possible autophagy) expressed at this 3-day time point.^12^ *CASP4* indicates Caspase 4; *CHAC1*, ChaC glutathione specific gamma-glutamylcyclotransferase1; *JMY*, Junction Mediating and Regulatory Prwotein; RT-qPCR, reverse transcription–quantitative polymerase chain reaction; *TNFRSF9*, Tumor Necrosis Factor; UPR, unfolded protein response.

Our microarray analyses identified more than 1000 unique transcripts that were up- or downregulated 2-fold or more in the treated compared with the control media grown MDA-MB-231 cells.^12^ *JMY* showed a significant upregulation in the treated versus control groups on microarray analyses while pro-apoptotic *CHAC1, TP531NP, TNFRSF9, ATF4, CASP4* and pro-survival *XBP1* also showed significant upregulation (Figure 6).^12^ We then conducted RT-qPCR on *CHAC1, TNFRSF9, XBP1, CASP4*, and *JMY* and analyzed the data with delta-delta Ct method and unpaired t-tests (Figure 7, Tables 1 and 2) and unpaired t-tests (Table 2).

**Table 2.** Unpaired t-tests of RT-qPCR for *JMY* in treated versus control MDA-MB-231 cells.

Gene	Unpaired t-tests			Mean/control	Mean/treated
	*df*	ts	*P*		
*JMY*	8	25.3296	.000000000682	9.7	7.4
*CHAC1*	8	26.796	.000000004	13.26134	6.184
*TNFRSF9*	8	26.7993	.0000000004	13.55	8.05
*CASP4*	8	7.6846	.0000583	8.214653	5.63
*XBP1*	8	12.7308	.00000136	5.74	4.34

Abbreviations: *CASP4* indicates Caspase 4; *CHAC1*, ChaC glutathione specific gamma-glutamylcyclotransferase1; *JMY*, Junction Mediating and Regulatory Protein; RT-qPCR, reverse transcription–quantitative polymerase chain reaction; *TNFRSF9*, Tumor Necrosis Factor; *XBP1*, X-box binding protein 1.

## Discussion

In our tubulin assay analyses, the data suggest round apoptotic fragmentation in a population of the cells that appears to be accompanied by microtubule-rich spikes that project through the cortex of the dying cells as well as a population of cells with irregular apoptosis that presents with the characteristics of autophagy-necrosis in the MDA-MB-231 cells when grown in treated versus control media (Figures 1 and 5; Tables 3 and 4).^15^ It has also been previously reported that the MDA-MB-231 cells were shown to display positive annexing staining at the 7-day time point which is also suggestive of apoptosis.^12^ The significant upregulation of *JMY* (Figures 6 and 7; Table 2) which is known to participate in transcriptional regulation, actin assembly, and membrane trafficking may be linked to the actin assembly/bundling required to promote microtubule organizational changes in both the observed possible rounded apoptosis (transcriptional regulation with possible unbranched actin bundling) and autophagosome formation (with membrane trafficking and possible branched actin bundling) suggestive of autophagy (irregular necrosis-like apoptosis) (Figures 1 and 5).^16^

**Table 3.** General characteristics of apoptosis versus necrosis.

Apoptosis	Necrosis
Programmed cell death	Premature cell death
Shrinking of the cytoplasm, nuclear condensation, DNA fragmentation	Swelling of the cytoplasm and nucleus followed by cell lysis
Caspase dependent	Caspase independent

**Table 4.** Possible indicators of apoptosis and necrosis noted in MDA-MB-231 cells.

Apoptosis indicators	Necrosis indicators
Upregulation of *CHAC1*	Enlargement of cytoplasm
Upregulation of *TNFRSF9*	Swelling of nucleus
Upregulation of *CASP4*	Cell membrane lysis
Upregulation of *TP531NP*	Nuclear degradation
Positive annexin staining	Microtubule disorganization
Microtubule spikes/rounding up	

Abbreviations: *CASP4* indicates Caspase 4; *CHAC1*, ChaC glutathione specific gamma-glutamylcyclotransferase1; *TNFRSF9*, Tumor Necrosis Factor; *TP531NP*, Tumor protein 53-induced nuclear protein 1.

### Round apoptosis (transcriptional regulation)

Oxidative stress and the initiation of apoptosis are known to spark cytoskeletal reorganization which involves association and dissociation (polymerization/depolymerization) of monomers from the filaments.^17^ The MDA-MB-231 showed a significant upregulation of ER stress and an activation of the rescue/repair pathway of the cell, the UPR.^12^ Significant upregulation of *CHAC1* and *CASP4* that are linked to the UPR suggests the MDA-MB-231 cells are initiating apoptosis (rounded/transcriptional regulation) (Tables 3 and 4).^12^ *TNFRSF9* also shows significant upregulation and is also known to regulate the apoptotic process.^18^ Here we note striking differences in the reorganization/elongation of the microtubules (microtubule spikes) in the BFA dc DEP EMF treated cancerous cells (Figures 1 and 5). This microtubule reorganization operates in close conjunction with actin that is driven by adenosine triphosphate (ATP)-dependent mechanisms that are associated with the G-actin/ATP molecules that drive critical concentrations and homeostasis of the actin filaments.^19^ The nucleotide binding site of monomeric actin is tightly associated with ATP (G-actin/ATP) in vivo.^19^ The structural polarity of the actin filament and the irreversible nature of ATP hydrolysis during the actin assembly have implications for the rate and direction of the filament growth at the opposite ends (positive/negative) of actin filaments.^19^ The actin filaments in the cytoskeleton continuously undergo turnover of subunits by addition/subtraction at their plus and minus ends. The critical concentration for the growth of the minus end is known to be higher than for the plus end in the actin filaments under general physiological conditions.^17^ Also, self-organization of microtubule networks have been initiated in vitro by combining tubulin, microtubule motors, and ATP.^19^ The BFA dc DEP-EMF’s influence on diamagnetic anisotropy via membrane potential, chloride ion channel modulation and significantly increased transcription (Figure 4)^14^ may possibly offer more available ATP (G-Actin/ATP complexes—thereby increasing critical concentrations high enough to affect both the plus and minus end of the actin [unbranched] filaments and subsequent reorganization/elongation of microtubules) to be used by the cell and facilitate cytoskeletal reorganizations (round apoptotic microtubule spikes) for the round apoptosis in these MDA-MB-231 cells (Figure 1).^20^

### Irregular necrosis-like apoptosis

While autophagy appears to play a significant role in cancer and other diseases and has been highly researched, it remains poorly understood.^21^ Autophagy has been seen as a survival response to delay apoptosis. But it has also been shown to promote irregular apoptotic necrosis-like cell death in cells with impaired apoptosis (*P53* deficient cells).^22^ Autophagy is also a cellular process that is regulated by signaling upstream of *MTORC1* and facilitates orderly degradation and recycling of cellular components that are stored in autophagosomes.^22^ Examples of signaling that can initiate this phenomena are energy signals controlled by AMP (activated protein kinase- sensor of cellular energy status)/ATP ratio, nutrient signals from growth factors or amino acids, and stress signals (ER stress, DNA damage, hypoxia, etc).^23^ Autophagosome formation to sequester the degraded cellular components requires a rapid recruitment of membrane to form the phagophore.^24^ Prolonged and excessive autophagosome formation leads to cytoplasmic and nuclear enlargement/swelling. Nuclear swelling has been noted to be a signal from the grave.^25^ There is evidence that the source of this recruited membrane needed for autophagosome formation originates from the endoplasmic reticulum (ER), Golgi, mitochondria, and plasma membranes.^24^ There is also evidence that shows the role of autophagy as both a tumor suppressor and in tumor survival. Starvation and ER stress response are linked to the autophagic protective processes and if the cell cannot recover from the stress, the cell initiates apoptosis (Figure 1).^26^ It has also been shown that prolonged ER stress can result in necrosis-like cell death with autophagy in cells that display impaired apoptosis (Figure 1F, G, and H). Also, inhibition of autophagy during ER stress has resulted in *reduced* cell death in apoptosis-deficient cells.^22^ It has been previously reported in these studies that ER stress appears to be a factor in the initiation of the UPR with strong transcriptional upregulation of the *ATF4-CHOP-CHAC1* apoptotic arm that coincides with the significant upregulation of *CASP4* in the treated MDA-MB-231 cells (Figures 4, 6, and 7).^12^ These MDA-MB-231 cells also show an increased gene expression of the survival arm of the UPR as seen in the significant upregulation of *XBP1* and the tubulin data suggest an increase in autophagy (survival/rescue mechanism) on days 2 and 6 (Figures 3, 6, and 7; Table 2).^12,27^ An increased availability of ATP, as opposed to the standard decreased energy/starvation factors associated with autophagy, may lead to prolonged ER stress in the setting of DNA damage (P53 deficient) in these BFA-treated MDA-MB-231 cells and possibly initiate excessive autophagosome formation, enlarged nuclei (and subsequent breakdown of nuclear envelope), extensive tubulin assembly/microfilament branched bundling (actin), cytoskeleton catastrophe, and ultimately irregular apoptotic-autophagy-necrosis-like cell death (Figures 1, 3, and 5; Tables 3 and 4).^25,28^ There appears to be a transition (Figure 3) from autophagy to apoptosis over time (due to possible prolonged ER stress) in these BFA-treated cells.

### JMY expression influence on round and irregular apoptosis

*JMY* is thought to play a dual role in cancer biology by having both a tumor suppressive capacity in the setting of DNA damage where it enhances *P53* activity and a tumor metastasis promoter due to its ability to nucleate actin filament.^29^ Our microarray analysis shows a significant and greater than 2-fold increase in *TP531NP* expression which suggests that the upregulation of *JMY* may have been modulating *P53* activity in this cell line under the influence of the BFA. *JMY’s* increased expression in our RT-qPCR also suggests this NPF’s possible influence in the cytoskeletal reorganization (nucleation of unbranched actin filaments to help facilitate the microtubule spikes and transport through the Golgi for phagosome formation or branched actin bundling in the cytosol) in both of the round apoptotic and the irregular autophagic-necrosis like MDA-MB-231 cells grown in the treated media. It is known that tubulin assembly and actin bundling that trigger cytoskeletal catastrophe and cell necrosis can occur.^30^ Also, a possible change in the AMP/ATP ratio (more available ATP) from the BFA influence, via chloride ion channel modulation (*_b_Cl*^−^) suggested by the strong increase in transcription noted in our microarray analyses in the treated versus control MDA-MB-231 cells could possibly offer a novel mechanism/application (instead of the historical starvation mechanism) for the possible autophagy/branched actin bundling and microtubule spikes/unbranched actin bundling noted in this cancerous cell line.^12,14,31^

## Conclusions

Triple-negative breast cancer remains a disease that is resistant to many current therapies.^4,5^ The ability of a certain population of this MDA-MB-231 cell line to strongly upregulate the survival mechanisms of the UPR (*XBP1*) and display autophagy in the presence of strong transcriptional apoptotic induction in a larger population of this cell line may speak to a possible explanation for the virulence of this disease. The BFA’s novel mechanisms of chloride ion channel and membrane potential modulation may offer hope for a future adjunct treatment for these diseased cells.^14^ These tumor cells that retain their apoptotic programming appear to respond to the noted pro-apoptotic transcriptional upregulation but the cells that respond with autophagy may indeed be apoptotic (*P53*) deficient and therefore able to survive the current apoptosis inducing treatments.^4,5^ The BFA may lead to an oxidative stress that is prolonged enough for these cells to transition from survival to an irregular necrosis-like apoptosis. In vivo modeling and future measures of the expressed protein levels are warranted to see how this interesting in vitro phenomena translates to the organism level.
